# Genome-wide association studies of leaf photosynthetic gas exchange parameters in sugarcane

**DOI:** 10.3389/fpls.2026.1792866

**Published:** 2026-05-07

**Authors:** Yongsheng Chen, Huiyi He, Xinglong Chen, Yinghua Shu, Yuanjiao Feng, Fengxiao Tan, Jianwu Wang

**Affiliations:** 1 College of Natural Resources and Environment, South China Agricultural University, Guangzhou, China; 2 Guangdong Sugarcane Genetic Improvement Engineering Center, Institute of Nanfan & Seed Industry, Guangdong Academy of Science, Guangzhou, China

**Keywords:** candidate genes, gas exchange parameters, GWAS, QTLs, sugarcane

## Abstract

**Introduction:**

As the global food crisis intensifies, improving photosynthesis is a primary strategy to enhance crop yield.

**Methods:**

The analysis was based on whole-genome sequencing data from updated abstract based on this version. 219 elite sugarcane clones of worldwide origin, grown across two sites over two years. The genetic bases of intercellular CO2 concentration (Ci), net photosynthetic rate (A), transpiration rate (E), and stomatal conductance (Gs) were investigated across four environments using 5,964,084 high-quality single nucleotide polymorphisms (SNPs).

**Results and discussion:**

A total of eight genomic intervals associated with gas exchange parameters were identified on six chromosomes (2A, 2C, 4B, 5B, 6A, and 8C), encompassing 10 significant SNPs within 20-kb windows. These SNPs showed P-values below 1.5 × 10^-6^ and were consistently detected in at least two environments. Among the eight predicted genes located within these intervals, three genes were identified as putative candidates affecting leaf photosynthesis. These genes are related to a WRKY transcription factor, the UV-B (Ultraviolet-B) receptor UVR8, and an R2R3 Myb protein. Overall, this study provides valuable molecular markers and candidate genes that could facilitate marker-assisted breeding for improved photosynthetic efficiency in sugarcane.

## 1 Introduction

Photosynthesis is the fundamental biological process sustaining most life on Earth and underpins global biomass production (Perera-Castro and Flexas, 2020). Each year, photosynthetic organisms convert more than 10^9^ metric tons of atmospheric CO_2_ into biomass. As the global population is expected to reach 9–10 billion by 2050, and food demand is rising, farmland is shrinking, and climate change is negatively impacting agricultural production, improving crop yield has emerged as a critical worldwide priority (Flügge et al., 2016). Improving photosynthetic efficiency, particularly the efficiency of light energy conversion, is therefore considered a promising strategy for increasing crop productivity in the long term (Flügge et al., 2016).

Photosynthetic gas exchange parameters, including intercellular CO_2_ concentration (Ci), net photosynthetic rate (A), transpiration rate (E), and stomatal conductance (Gs), represent integrative physiological traits that reflect the performance of the photosynthetic apparatus. These traits are widely used to evaluate photosynthetic capacity and to gain insight into the regulation and functioning of photosynthetic processes across environments (Wang et al., 2020a; Galeriani et al., 2022; Shamim et al., 2022), making them suitable targets for genetic analysis.

Genome-wide association studies (GWAS) provide a powerful approach for dissecting the genetic basis of complex quantitative traits by identifying associations between phenotypic variation and genome-wide genetic variants. Although GWAS was initially developed in human genetics, their experimental design has been widely adopted in plant research and has proven effective for identifying loci associated with agronomic (Baek et al., 2025; Li et al., 2025), quality-related (Nawade et al., 2025; Sunvittayakul et al., 2025), and stress-resistance (Zhang et al., 2025a) traits.

In crops, GWAS has been increasingly applied to investigate the genetic architecture of photosynthetic and photosynthesis-related traits. Numerous quantitative trait loci (QTLs) associated with photosynthetic gas exchange parameters have been reported across multiple environments in species such as rice (Wang et al., 2020a), maize (Zhang et al., 2017; Yi et al., 2023), soybean (Lü et al., 2018; Yang et al., 2020; Wang et al., 2020b; Shamim et al., 2022) and sorghum (Ortiz et al., 2017). However, despite the identification of many associated loci, the causal genes underlying these QTLs remain largely unresolved (Keller et al., 2024). To date, only a limited number of functionally validated candidate genes have been reported. Notably, GmFtsH25, encoding an FtsH-type protease, was recently identified in soybean, and its overexpression resulted in increased net photosynthetic rate, enhanced starch accumulation, and improved yield-related traits (Wang et al., 2023). This example highlights both the potential and the current limitations of GWAS in elucidating the molecular basis of photosynthetic traits.

Sugarcane (*Saccharum* spp.) is the world’s most harvested crop by tonnage (Healey et al., 2024) and plays a critical role in global sugar and bioenergy production (Wei et al., 2024). As a C_4_ grass, sugarcane exhibits exceptionally high photosynthetic efficiency and biomass accumulation compared with many other crop species (Dinesh Babu et al., 2022).

Despite its agronomic importance, genetic studies in sugarcane have long been constrained by the highly polyploid and aneuploid nature of its genome (Healey et al., 2024), which has complicated genome sequencing, assembly, and annotation efforts. Consequently, progress in understanding the genetic basis of complex traits in sugarcane has lagged behind that of other major crops.

Recent advances in sugarcane genomics have begun to address these limitations, with the publication of genome sequences for commercial hybrids such as R570 (Garsmeur et al., 2018; Healey et al., 2024), ZZ1 (Bao et al., 2024) and XTT22 (Zhang et al., 2025b), as well as for *S. spontaneum* accessions with 32 chromosomes (AP85-441) (Zhang et al., 2018) and 40 chromosomes (Np-X) (Zhang et al., 2022). These resources have enabled the application of GWAS to sugarcane, leading to the identification of loci associated with a range of traits, including cane yield (Zhang et al., 2023; Li et al., 2024), sucrose content (Zhang et al., 2023; Li et al., 2024), agronomic characteristics (Zhang et al., 2023; Li et al., 2024; Chen et al., 2025a), fiber content (Chen et al., 2025b), disease resistance (Chen et al., 2025c), and leaf angle (Chen et al., 2022). However, with the exception of the rust resistance locus *Bru1* (Parco et al., 2014; Li et al., 2024), few identified loci have been widely translated into breeding practice, and studies focusing on the genetic basis of photosynthesis-related traits in sugarcane remain scarce.

In our previous work, whole-genome sequencing of 219 elite sugarcane clones of worldwide origin yielded approximately six million high-quality single nucleotide polymorphisms (SNPs), providing a valuable resource for genome-wide analyses (Chen et al., 2022; Li et al., 2024; Chen et al., 2025b). Using this population, GWAS identified numerous SNPs associated with important agronomic traits, and the candidate gene *ShN/AINV3.1* was shown to positively influence stalk number, plant height, and stalk diameter (Li et al., 2024).

Building on these genomic resources, the present study employed SNP-based GWAS in the same panel of 219 elite sugarcane clones to identify loci controlling leaf photosynthetic gas exchange parameters across multiple environments. The objectives were to characterize the genetic variation underlying these traits, identify informative molecular markers and candidate genes, and provide a foundation for future functional studies and marker-assisted breeding aimed at improving photosynthetic efficiency in sugarcane.

## 2 Materials and methods

### 2.1 Sugarcane materials and field experiments

A sugarcane association panel consisting of 219 accessions was evaluated in field experiments conducted in 2019 and 2020 at two experimental sites of the Institute of Nanfan & Seed Industry, Guangdong Academy of Sciences: the Wengyuan base (24.36°N, 114.13°E; altitude 120 m) and the Zhanjiang base (21.39°N, 110.24°E; altitude 25 m). The 219 clones were collected worldwide from many sugarcane-producing countries. Among the tested clones, 18 were backbone parents, including CP28-11-Sanya-Yacheng, CP49-50, CP67-412, CP72-1210, Co1001, Co419, F108-Sanya-Yacheng, F134, Hua_nan-56-12, Hua_nan-56-21-Sanya-Yacheng, KeWu, NCo310, Neijiang-57-416, ROC1-Sanya-Yacheng, Yacheng-71-374, Yun-65-225, gui11, and yuenong-73-204. These backbone parents descended from remote ancestral parents of two to four species of *S.officinarum*, *S.spontaneum*, *S.barberi* and *S.robustum.* Additionally, three of these core parent materials—CP49-50, Co419, and NCo310—were traditional elite varieties that were used commercially worldwide. The remaining clones were bred from these backbone parents.

The 219 clones were planted, at the Wengyuan base on 26 February 2019, and at the Zhanjiang base on 18 February 2020. At both locations, the experiments followed a randomized complete block design with three replicates. Phenotypic data collection took place from 2022 to 2023 at two locations: at the Wengyuan base (from late July to early August 2022; from late June to early July and in early September 2023), and at the Zhanjiang base (in late September 2023).

At each site, each clone was planted in three rows in each replicate (i.e., each plot), with a row spacing of 110 cm. Each row contained 16 plants spaced 25 cm apart. Planting materials were disinfected and cut into single-bud setts for each clone. These setts were initially established in seedling nurseries and subsequently transplanted to the field when bud height reached approximately 20 cm. Field management practices at both sites followed standard local agronomic procedures for sugarcane cultivation, including fertilization, irrigation, and the control of diseases, pests, and weeds.

### 2.2 Phenotypic data collection and statistical analysis

Four photosynthetic gas exchange parameters, including intercellular CO_2_ concentration (Ci), net photosynthetic rate (A), transpiration rate (E), and stomatal conductance (Gs), were measured using a portable photosynthesis system (LCi-T, UK; Beijing Ecotech Ecological Technology Ltd.). Measurements were performed on sunny days from 09:00 to 11:00 a.m. The photosynthetic photon flux density was set to 1000 μmol m^-^² s^-^¹ using a light-emitting diode (LED) light source, while the CO_2_ concentration within the measurement chamber was not artificially controlled.

During sugarcane growth, gas exchange parameters were measured on the +2 leaf of five consecutive, normally growing plants located in the middle of each plot. Measurements were conducted across four environments, designated as H1 (Wengyuan base, late June to early July 2023), H2 (Wengyuan base, early September 2023), H3 (Zhanjiang base, late September 2023), and H4 (Wengyuan base, late July to early August 2022).

Normality tests, using the Shapiro-Wilk test, Bartlett test, and Levene test in combination with Quantile–quantile (Q–Q) plots, were first applied to the phenotypic data for all four traits. When significant or highly significant differences were detected, Wilcoxon rank-sum tests were used to compare trait values between pairs of environments. Correlation analyses were performed using the Pearson method implemented in R software (version 4.3.2; www.r-project.org), and all graphical visualizations were generated using the same software.

### 2.3 Single nucleotide polymorphism genotyping and population structure analysis

Whole-genome resequencing and SNP genotyping procedures have been described in detail in previous studies (Chen et al., 2022; Li et al., 2024; Chen et al., 2025b). Paired-end sequencing libraries and sequencing were performed on an Illumina Hiseq2500 platform by Beijing Novogene Bioinformatics Technology Co., Ltd. (Beijing, China), with an average sequencing depth of approximately 21× coverage based on *S. spontaneum*. After adapter sequences and low-quality sequences were first removed from the paired-end raw reads of each sample, the resulting cleaned reads were then mapped to AP85–441 with default parameters via bwa software. The uniquely mapped reads with correct insert lengths were retained for SNP calling by GATK (v4.1). SNPs were filtered using the following parameters: call quality divided by depth (QD) <2.0; mapping quality (MQ) <40.0; Fisher’s exact test (FS) >60.0; SOR (Strand Odds Ratio) >3.0; MQRankSum <-12.5; and ReadPosRankSum <-8.0. Then, using PLINK software (version 1.07), the SNPs were filtered with a minor allele frequency (MAF) greater than 0.05, and a missing rate lower than 0.2, and removing SNPs with more than two alleles. Finally, 5,964,084 high-quality SNPs were obtained.

Population structure was inferred using admixture software (version 1.3.0; https://vcru.wisc.edu/simonlab/bioinformatics/programs/#admixture). Principal component analysis (PCA) and kinship matrix (K matrix) estimation were performed using TASSEL 5.0 based on the above mentioned 5,964,084 high-quality SNPs.

The significance of principal components was evaluated using EIG-6.1.4, with the following parameters:/bin/twstats -t twtable -i pca.eigenval -o pca_number (Patterson et al., 2006; Zheng and Weir, 2016; Zhao et al., 2018).

Linkage disequilibrium (LD) was estimated using 5,964,084 SNPs, and LD (r²) was calculated using PLINK software (version 1.07) with the parameters --ld-window 99999 --ld-window-kb 500 --r2 --ld-window-r2 0 (Purcell et al., 2007).

### 2.4 Genome wide association study

Genome-wide association studies were conducted using the Gemma software package (version 0.98.5) (Zhou and Stephens, 2012). The association tests were performed using univariate linear mixed models without a covariate matrix, based on genotypic data comprising 5,964,084 SNPs obtained from the resequencing of the 219 sugarcane accessions. The relatedness matrix was calculated using the command:./gemma-bfile [prefix] -gk 2 -o [prefix], and association analyses were conducted using./gemma-bfile [prefix] -k [filename] -lmm 1 -o [prefix]. The significance threshold was set at *P* = 1.5 × 10^-6^, consistent with previous studies (Li et al., 2024). Manhattan and Quantile–Quantile (Q–Q) plots were generated using the “CMplot” package in R software (version 4.3.2; www.r-project.org).

### 2.5 Candidate gene identification

**Figure 1:**
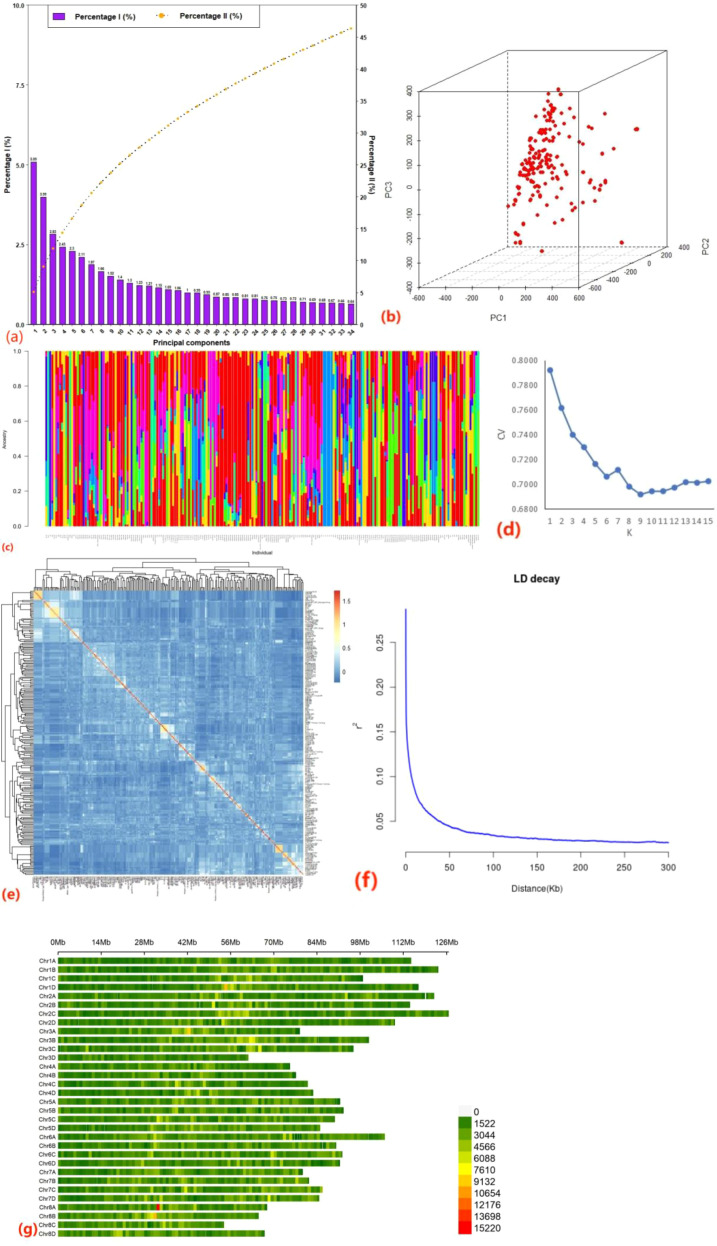
Population structure, principal component analysis, kinship, linkage disequilibrium, and SNP distribution of the 219 sugarcane accessions. **(a)** Percentage of variance explained by individual principal components (PCs) and cumulative variance explained by the first 34 PCs. **(b)** Three-dimensional scatterplot of the first three principal components. **(c, d)** Population structure inferred using ADMIXTURE, showing nine subgroups based on the optimal K value. **(e)** Heatmap of pairwise kinship coefficients among accessions. **(f)** Genome-wide linkage disequilibrium (LD) decay. **(g)** Distribution of SNP numbers across chromosomes. Different color represented the number of SNPs within 1Mb window size.

To identify potential candidate genes related to photosynthetic gas exchange traits, genomic regions extending 20 kb upstream and downstream of each SNP peak were defined as candidate intervals, based on the observed LD decay distance (**Figure 1f**), which showed that the decay rate was rapid, with an LD distance of approximately 20 kb when r^2^ decreased to 0.1. This window size was consistent with that used in previous sugarcane GWAS analyses (Li et al., 2024; Chen et al., 2025b). Genes located within the intervals were classified as candidate genes, and annotation information was obtained for all genes containing associated markers.

## 3 Results

### 3.1 Variation and correlation of gas exchange parameters

**Figure 2:**
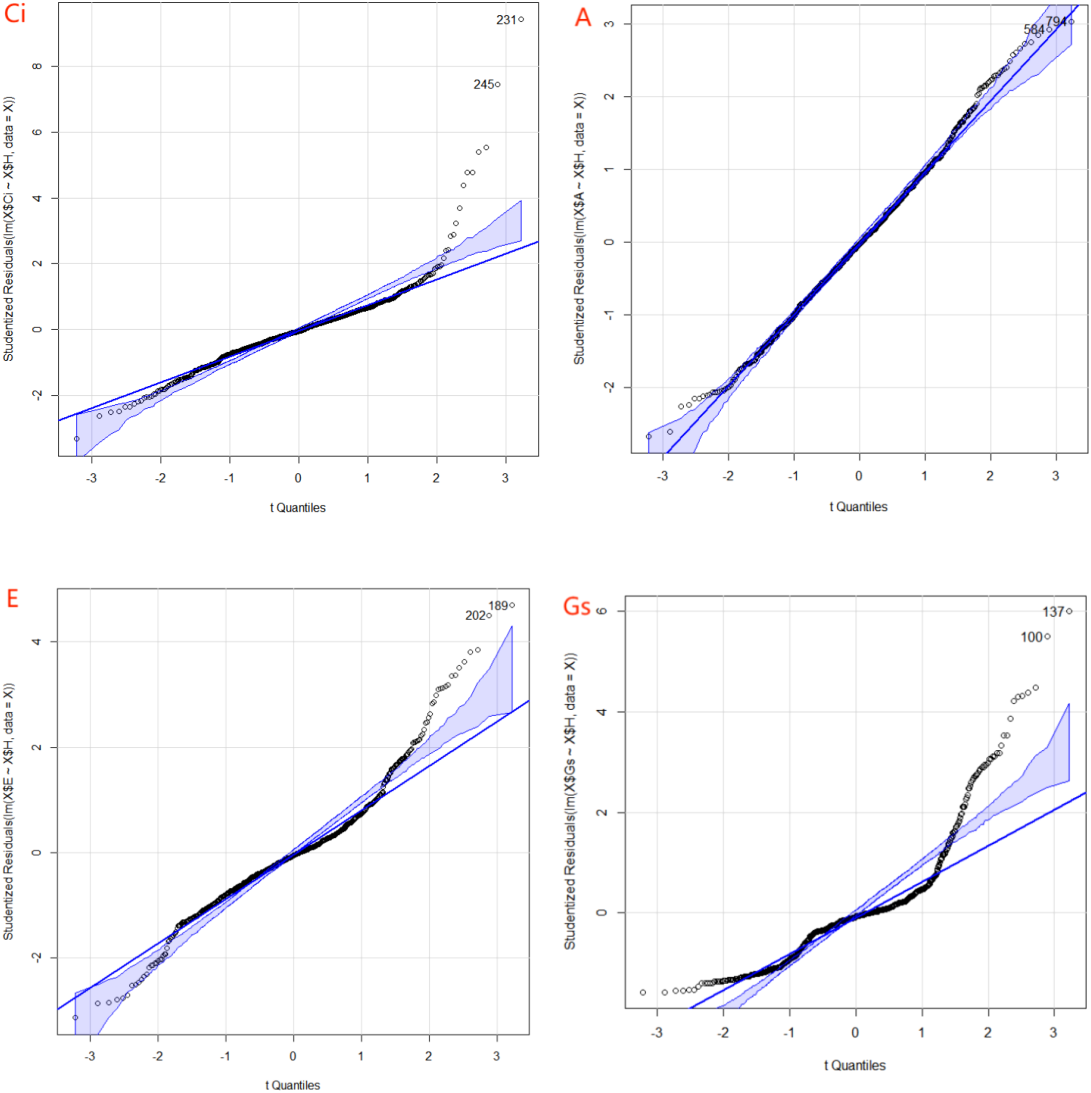
Quantile–quantile (Q–Q) plots showing the results of normality tests for phenotypic data. The four gas exchange parameters include intercellular CO_2_ concentration (Ci), net photosynthetic rate (A), transpiration rate (E), and stomatal conductance (Gs). Phenotypic data were collected across four environments: H1 (Wengyuan base, late June to early July 2023), H2 (Wengyuan base, early September 2023), H3 (Zhanjiang base, late September 2023), and H4 (Wengyuan base, late July to early August 2022).

**Figure 3:**
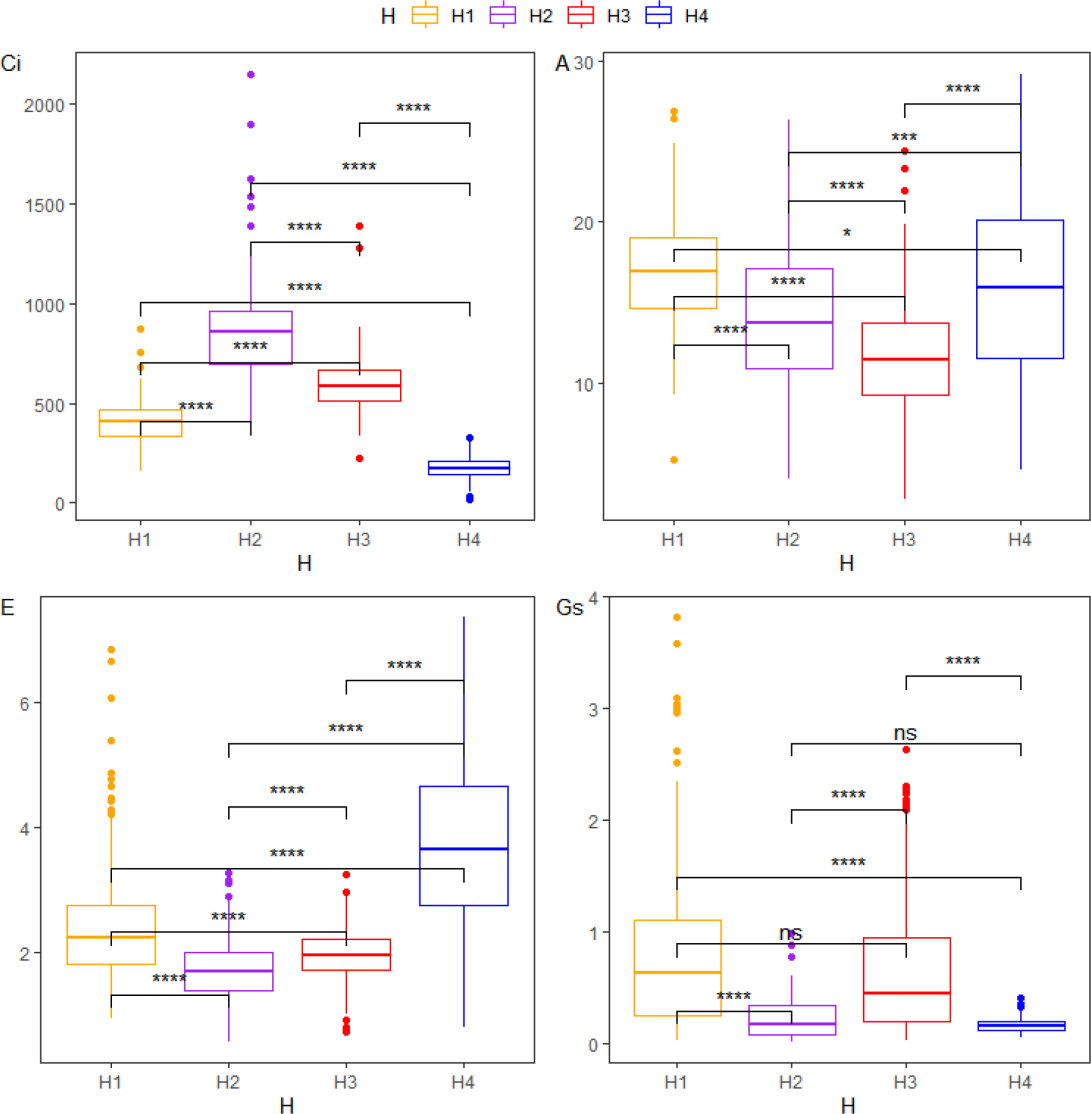
Boxplots showing the distribution of the four gas exchange parameters (Ci, A, E, and Gs) across the four environments, and pairwise comparisons of trait values between environments based on Wilcoxon tests. The four gas exchange parameters include intercellular CO_2_ concentration (Ci), net photosynthetic rate (A), transpiration rate (E), and stomatal conductance (Gs). Phenotypic data were collected across four environments: H1 (Wengyuan base, late June to early July 2023), H2 (Wengyuan base, early September 2023), H3 (Zhanjiang base, late September 2023), and H4 (Wengyuan base, late July to early August 2022). *p < 0.05, ***p < 0.001, ****p < 0.0001; ns, no significance (Wilcoxon rank-sum tests).

With the exception of a single Shapiro-Wilk test for A (P < 0.01), all Shapiro-Wilk, Bartlett, and Levene tests for each trait across the four environments resulted in P-values < 0.001 (**Table 1**). Normality tests, combining the Shapiro-Wilk, Bartlett, and Levene tests with Q-Q plots, indicated that the phenotypic data for all four gas exchange parameters deviated from a normal distribution across environments (**Figure 2**). Substantial variation was observed among the 219 sugarcane accessions for all traits, and Wilcoxon tests revealed highly significant differences for each trait between pairs of environments (**Figure 3**; **Supplementary Tables 1**, **S2**). Similar extensive variation in gas exchange parameters across genotypes, environments, or developmental stages has been reported in previous studies (Zhang et al., 2017; Yang et al., 2020; Keller et al., 2024).

**Table 1:** Results of the Shapiro-Wilk, Bartlett, and Levene tests for the phenotypic data of photosynthetic gas exchange parameters of the sugarcane association panel comprising 219 accessions across four environments.

Trait	W (Shapiro-Wilk test)	K-squared (Bartlett test)	F (Levene test)
Ci (ppm)	0.94***	342.54***	46.73***
A (umolCO_2_ m^-2^ s^-1^)	0.99**	59.11***	24.49***
E (mol m^-2^ s^-1^)	0.86***	331.22***	74.56***
Gs (mol m^-2^ s^-1^)	0.70***	930.49***	78.68***

**p < 0.01, ***p < 0.001.

The data showed that the four gas exchange parameters displayed distinct patterns across all environments. For intercellular CO_2_ concentration (Ci), H2 had the highest mean value, followed by H3, H1, and H4 (lowest). The net photosynthetic rate (A) was highest in H1, followed by H4, H2, and H3 (lowest). H4 exhibited the highest mean transpiration rate (E), followed by H1, H3, and H2. Stomatal conductance (Gs) was highest in H1, then H3, with H2 and H4 showing the lowest mean values. Overall, the early growth stages (H1 and H4) showed higher net photosynthetic rate (A) and transpiration rate (E) than the later stages (H2 and H3) (**Supplementary Table 2**, **Figure 3**). This indicates that sugarcane likely has higher photosynthetic efficiency in its early stages compared to later ones.

**Figure 4:**
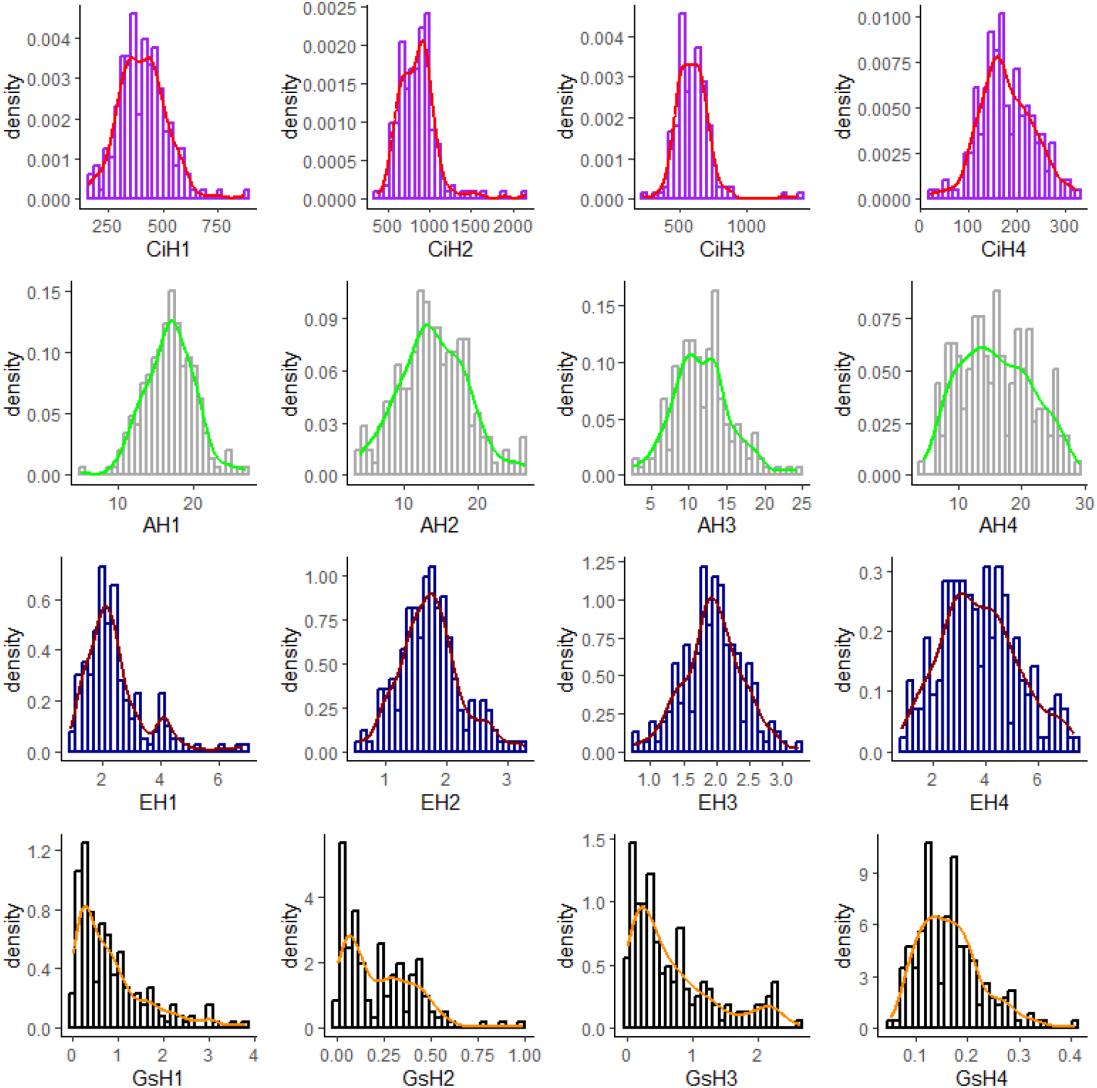
Density distributions of the four photosynthesis-related gas exchange parameters (Ci, A, E, and Gs) measured in the 219 sugarcane accessions across the four environments. The four gas exchange parameters include intercellular CO_2_ concentration (Ci), net photosynthetic rate (A), transpiration rate (E), and stomatal conductance (Gs). Phenotypic data were collected across four environments: H1 (Wengyuan base, late June to early July 2023), H2 (Wengyuan base, early September 2023), H3 (Zhanjiang base, late September 2023), and H4 (Wengyuan base, late July to early August 2022). The red, green, brown and orange lines all represented the smooth distribution of the four gas exchange parameters in the corresponding environment, which followed either a continuous normal or skewed pattern.

The skewness and kurtosis values indicated that the distributions of all four gas exchange parameters were closer to normal in H4 than in the other three environments. In contrast, the distributions of Gs across all four environments were more skewed than those of the other three parameters. Meanwhile, the distributions of Ci, A, and E were closer to normal in H4, H2, and H3, respectively (**Supplementary Table 2**, **Figure 4**).

Overall, the distributions of all four traits were continuous and approximately normal or skewed across environments, indicating that they are quantitative traits likely controlled by multiple genes and suitable for genome-wide association analysis (**Supplementary Table 2**, **Figure 4**).

**Figure 5:**
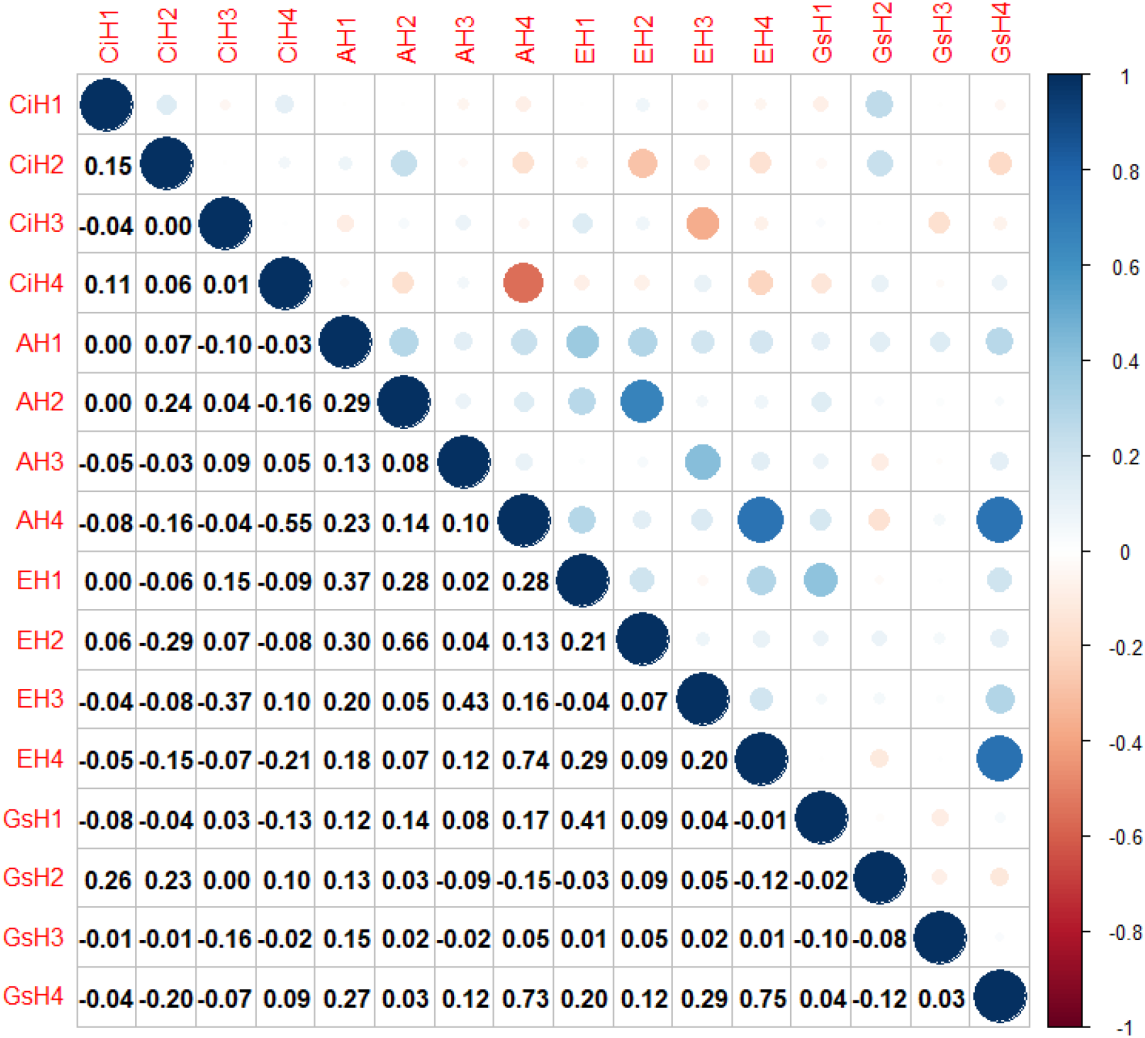
Visualization of Pearson correlation coefficients among the four gas exchange parameters (Ci, A, E, and Gs) in the 219 sugarcane accessions across each environment. The four gas exchange parameters include intercellular CO_2_ concentration (Ci), net photosynthetic rate (A), transpiration rate (E), and stomatal conductance (Gs). Phenotypic data were collected across four environments: H1 (Wengyuan base, late June to early July 2023), H2 (Wengyuan base, early September 2023), H3 (Zhanjiang base, late September 2023), and H4 (Wengyuan base, late July to early August 2022).

Pearson correlation analysis revealed consistent relationships among gas exchange parameters across environments. Overall, A, E, and Gs were positively correlated with one another, whereas Ci showed negative correlations with A, E, and Gs. These relationships were particularly pronounced in environment H4, where strong positive correlations were observed among A, E, and Gs (correlation coefficients ranging from 0.73 to 0.75), while Ci was significantly and negatively correlated with A (r = −0.55) (**Figure 5**).

### 3.2 Population structure, principal component analysis, and kinship

Significance testing of principal components showed that the first 34 PCs explained a significant proportion of the phenotypic variance; however, their cumulative contribution remained below 50% (**Figure 1a**). Population structure analysis using ADMIXTURE suggested an optimal subdivision of the 219 accessions into nine subgroups (**Figures 1c, d**). Despite this, no pronounced population stratification was evident, as supported by the three-dimensional scatterplot of the first three PCs (**Figure 1b**) and the kinship heatmap (**Figure 1e**). Analysis of SNP distribution revealed that chromosome 2C contained the largest number of SNPs (252,702), whereas chromosome 3D contained the fewest (107,559), with an average of 186,378 SNPs per chromosome. Moreover, the lengths for these 32 chromosomes ranged from 53.63 Mb to 126.61 Mb, and the coverage density of these SNP markers ranged from 381.05 bp to 597.15 bp with an average of 485.95 bp (**Figure 1g**). These results indicate a relatively even genomic coverage and limited confounding population structure, supporting the suitability of the population for GWAS.

### 3.3 Genome-wide association study

Genome-wide association analysis was performed using 5,964,084 high-quality SNPs and phenotypic data for the four gas exchange parameters across four environments, employing a univariate linear mixed model implemented in GEMMA (v.0.98.5) (Zhou and Stephens, 2012). As mentioned above, the cumulative contribution of the first 34 PCs, which explained a significant proportion of the phenotypic variance, remained below 50% (**Figure 1a**). No pronounced population stratification was evident, as supported by the three-dimensional scatterplot of the first three PCs (**Figure 1b**) and the kinship heatmap (**Figure 1e**), where no obvious block-like clusters among these clones were observed. Therefore, no covariate matrix was included, as population structure analyses indicated no obvious stratification among accessions.

The present investigation of the association panel (planted in 2019 and 2020) was conducted from 2022 to 2023. However, some materials gradually died after years of ratoon cultivation in certain environments. During the present analysis, association test with univariate linear mixed models was performed for genotypic data of each trait in each environment. The total number of the association tests was 16 (four traits across four environments). Specifically, 20 of the 219 tested clones were excluded from all 16 tests because they had died across all four environments, resulting in no available data for any trait (**Supplementary Table 1**). For the remaining missing data, the corresponding clones had died in only one specific environment, with the exceptions of one clone that had died in three environments and two clones that had died in two environments.

**Figure 6:**
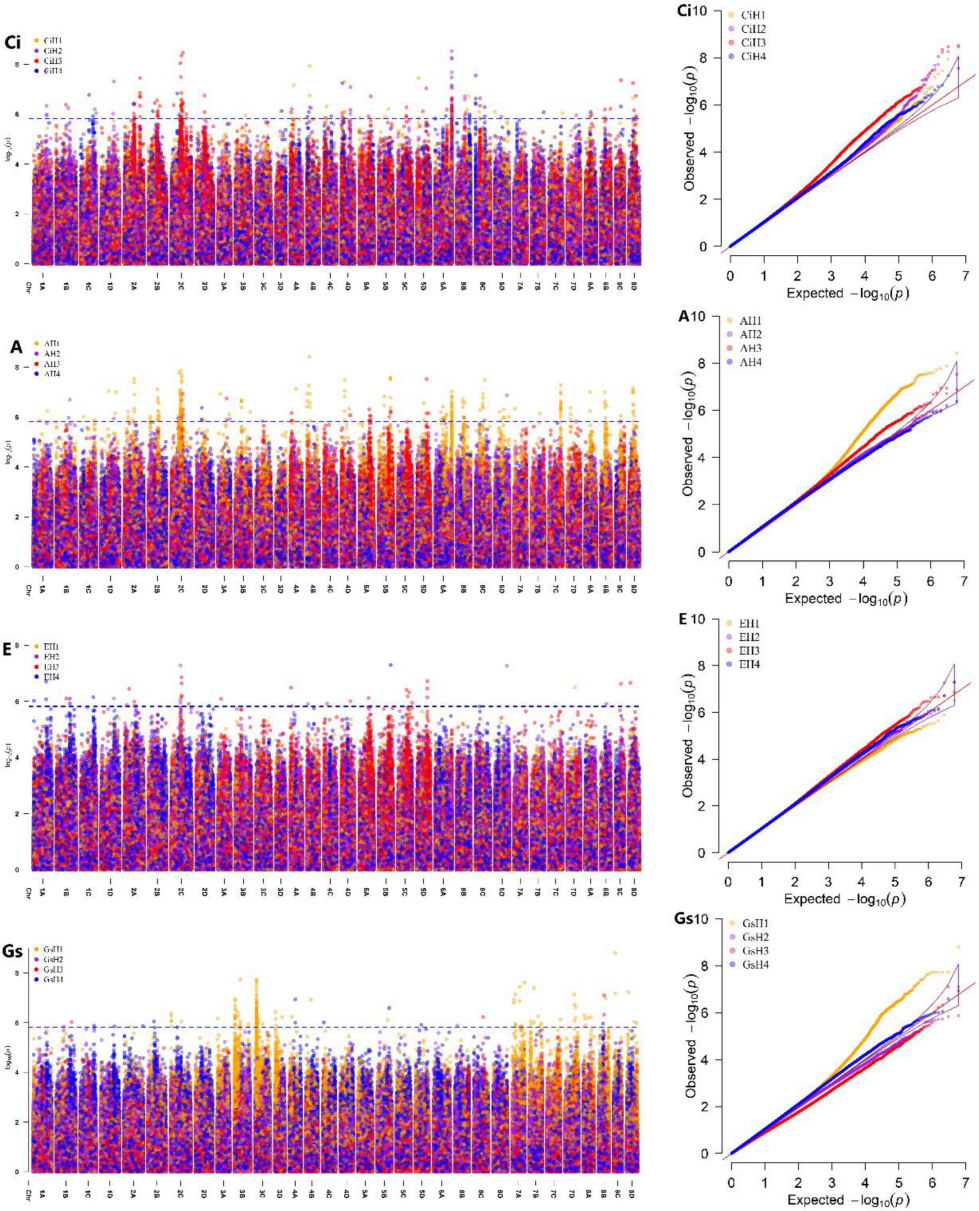
Manhattan plots and corresponding quantile–quantile (Q–Q) plots for genome-wide association analyses of intercellular CO_2_ concentration (Ci), net photosynthetic rate (A), transpiration rate (E), and stomatal conductance (Gs) across four environments. Phenotypic data were collected across four environments: H1 (Wengyuan base, late June to early July 2023), H2 (Wengyuan base, early September 2023), H3 (Zhanjiang base, late September 2023), and H4 (Wengyuan base, late July to early August 2022). The blue dashed lines all represented the significance threshold of p = 1.5 × 10^−6^.

A total of 10 significant SNPs were identified as being associated with gas exchange parameters. Significance was defined as SNPs showing P-values below 1.5 × 10^-6^ for the same trait in at least two environments. Among these 10 SNPs, seven associated with Ci were detected on chromosomes 2A, 2C, 4B, 6A, and 8C, with phenotypic variance explained (PVE) ranging from 11.21% to 14.57%. Four associated with A were identified on chromosomes 2C, 5B, 6A, and 8C, explaining 10.35% to 12.35% of the phenotypic variance. One SNP (Chr8C_32741161) was associated with both Ci (PVE = 12.83%) and A (PVE = 10.49%). No significant SNPs were detected for E or Gs, possibly reflecting stronger environmental influences on these traits. These SNPs were distributed across six chromosomes: 2A, 2C, 4B, 5B, 6A, and 8C, and across eight intervals, with two of the intervals each containing two significant SNPs (**Figure 6**; **Supplementary Tables 3, S4**).

### 3.4 Candidate gene analysis

Based on the significant SNPs, eight genomic intervals were defined using a 20-kb window on either side of each SNP peak. Four of these intervals were located within annotated genes, while the remaining intervals were intergenic. In total, eight genes were predicted within these regions based on the *Saccharum spontaneum* L. reference genome (AP85-441) (Zhang et al., 2018) (**Supplementary Table 4**). Among these, three genes were considered potential candidates influencing leaf photosynthesis based on their functional annotations and previous studies, and they were *Sspon.02G0019170-1A*, *Sspon.02G0019190-3C*, and *Sspon.04G0000680-2B*.

## 4 Discussion

### 4.1 The *Saccharum spontaneum* L. reference genome and this association panel

In this study, genome-wide association analysis of leaf photosynthesis-related traits was conducted using a panel of 219 elite sugarcane clones whose whole genomes were sequenced in 2019, when the association panel was planted at the Wengyuan site in Guangdong Province, China. At that time, sequencing reads were aligned to the *Saccharum spontaneum* L. reference genome (AP85-441), which had been published in 2018 (Zhang et al., 2018). Reference genomes for the commercial sugarcane hybrids R570 (Healey et al., 2024) and ZZ1 (Bao et al., 2024) have only recently become available. In contrast, our previous GWAS analyses, all of which were based on the *S. spontaneum* genome, successfully identified loci associated with multiple traits, including fiber content (Chen et al., 2025b), sucrose content, stalk number, plant height, stalk diameter, cane yield, sugar yield (Li et al., 2024) and leaf angle (Chen et al., 2022). The present study builds directly on this foundation by further dissecting the genetic basis of leaf photosynthetic gas exchange parameters using the same well-characterized association panel. Therefore, the present analysis was necessarily based on AP85–441 to ensure methodological consistency with our earlier work, which represents a direct extension of this established research framework. Additionally, based on AP85-441, SNPs associated with brix, stalk height, stalk number, stalk diameter, and cane yield were identified in Guangxi, China (Zhang et al., 2023). Since 2022, only two reports about GWAS based on LCP 85–384 and R570 genomes were respectively in 2023 (Zhang et al., 2023) and 2025 (Chen et al., 2025a); and up to now, among the only two reports about GWAS based on XTT22 genomes both in 2025 (Zhang et al., 2025b; Chen et al., 2025c), one was just that we identified SNPs associated with sugarcane smut resistance in the same association panel (Chen et al., 2025c). Thus, the *S. spontaneum* genome remains a valuable and widely used reference for sugarcane genomic studies. In contrast, based on XTT22 genome and in the same association panel, we identified 68 loci significantly associated with sugarcane smut resistance across seven environments by GWAS in 2025, however, of these, 44 loci were respectively identified from certain one environment and an additional 24 loci were identified using the BLUP values, integrating all phenotypic datasets, and no loci were detected across two or more environments (Chen et al., 2025c). Conversely, these 10 significant SNPs based on AP85–441 in present study were all detected in at least two environments for the same trait.

GWAS results are known to be influenced by the composition and structure of the association population, even when the same reference genome is used. Differences in genetic background, allele frequency, and environmental adaptation can result in the detection of distinct significant SNPs or QTLs for the same trait across populations. For instance, using the same reference genome, previous GWAS conducted in Guangxi Province using a population of 159 sugarcane genotypes identified SNPs associated with brix, stalk height, stalk number, stalk diameter, and cane yield (Zhang et al., 2023) that differed from those detected in our studies using the 219 elite clones evaluated in Guangdong Province (Li et al., 2024). These observations highlight the population-specific nature of GWAS signals and emphasize the importance of validating loci across diverse genetic backgrounds and environments.

**Figure 7:**
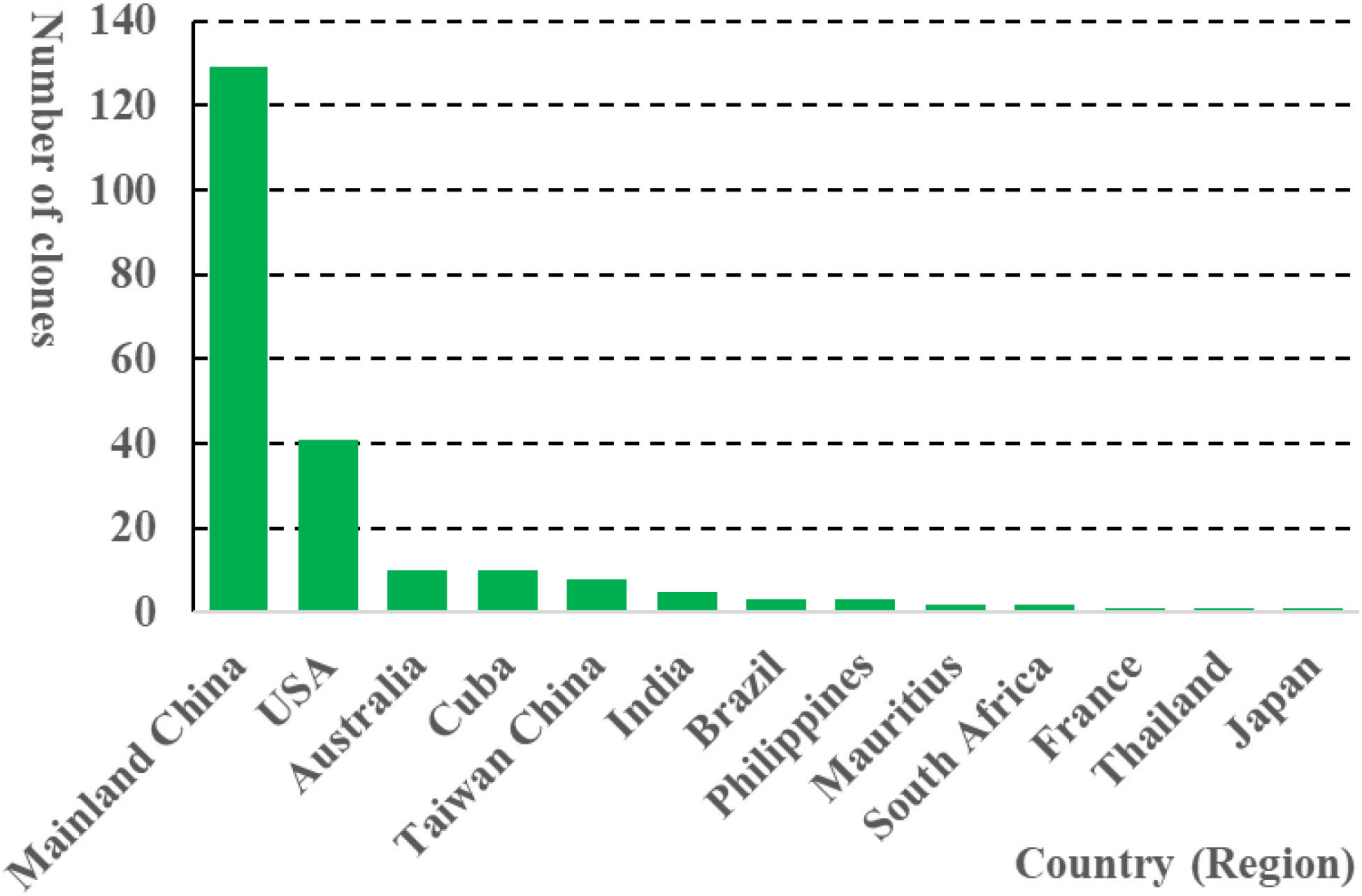
Histogram of the distribution of the countries or regions which the tested clones were derived from.

In this study, the tested 219 clones were derived from 13 countries or regions, with the exception of three whose origins were unclear. They were mainly from Mainland China (129) and the USA (41), while the number of clones from the other eleven countries or regions was fewer than 10 (**Figure 7**). Among the 219 clones, 19 were released before 1959, 103 between 1960 and 1999, 86 after 2000. Additionally, 11 clones lacked specific breeding year information (Li et al., 2024). Previous GWAS conducted in Guangxi Province identified SNPs associated with brix, stalk height, stalk number, stalk diameter, and cane yield using a population of 159 genotypes, which derived from hybrids by crossing and were obviously divided into four subgroups with 12, 7, 50, and 90 genotypes (Zhang et al., 2023). In contrast, there was no obvious stratification among the present accessions, and the reasons could be that this population covers a wide range of genetic diversity of sugarcane and is not significantly influenced by artificial selection or geographical isolation. These results suggest that​ the present population could more accurately reflect the natural genetic variation of sugarcane, providing a more applicable reference for subsequent genetic research and breeding applications.

### 4.2 Photosynthetic gas exchange parameters and these SNP markers

In this study, relatively weak correlations were observed among some photosynthetic gas exchange parameters across environments. This may reflect the strong environmental sensitivity of these traits, as gas exchange measurements are typically obtained over short time windows and may not fully capture photosynthetic performance across the entire growing season (Keller et al., 2024). Photosynthetic gas exchange parameters, including intercellular CO_2_ concentration, net photosynthetic rate, transpiration rate, and stomatal conductance, are complex and dynamic traits regulated by multiple physiological processes and environmental factors (Zhang et al., 2017). As a result, stable correlations among these traits are not always observed under varying field conditions.

Previous studies have shown that the relationships among photosynthesis-related traits can vary depending on environmental conditions. For instance, the correlation between net photosynthetic rate and stomatal conductance was reported to be relatively weak under normal phosphorus conditions but substantially stronger under low phosphorus stress, suggesting that environmental constraints may enhance the coordination among photosynthetic traits (Yang et al., 2020). Consistent with this observation, stronger correlations among gas exchange parameters were detected in environment H4 compared with the other three environments in the present study. These findings further support the notion that environmental context plays a critical role in shaping the physiological relationships among photosynthetic traits and influences their genetic detectability in GWAS.

In this study, 5,964,084 high-quality SNPs were screened and covered the full length of 2898.23 Mb of 32 chromosomes. The average coverage density was 485.95 bp for these SNP markers, which was significantly less than the LD distance of approximately 20 kb when r^2^ decreased to 0.1. It is practicable to employ this SNP-based GWAS.

### 4.3 Key candidate genes

Based on the functional annotations and previous studies, three key candidate genes were considered potential ones influencing leaf photosynthesis.

The candidate gene *Sspon.02G0019170-1A* on chromosome 2A, associated with Ci, encodes a WRKY transcription factor. WRKY proteins constitute a large family of plant transcription factors involved in diverse physiological processes, including responses to biotic and abiotic stresses and regulation of plant development (Satapathy et al., 2018; Rashid et al., 2023). WRKY transcription factors can be involved in phytohormone-mediated signaling pathways by acting as downstream target genes or regulating phytohormone anabolism to modulate plant responses to biotic stresses (Wang et al., 2024). It is reported that overexpression of *AtWRKY6*, *AtWRKY18*, *AtWRKY53* or *AtWRKY70* generally resulted in small, stunted transgenic plants, and nearly all such lines showed altered leaf morphologies (Ülker and Somssich, 2004). Transcriptomic analyses have shown that members of the *SsWRKY* gene family exhibit diverse temporal and spatial expression patterns in *S. spontaneum*, with several genes implicated in photosynthesis-related processes (Li et al., 2020; Wang et al., 2024). Among them, *SsWRKY76* and *SsWRKY145* inhibit expression of the light-harvesting chlorophyll *a/b*-binding proteins; *SsWRKY21* and *SsWRKY22* regulate light-dependent stomatal opening in guard cells (Li et al., 2020).

Another candidate gene, *Sspon.02G0019190-3C* on chromosome 2C, also associated with Ci, encodes UVR8, an UV-B (Ultraviolet-B) receptor known to regulate phototropism, chloroplast development, stomatal opening, and leaf development (Yin and Ulm, 2017; Chen et al., 2021). High doses of UV-B can affect plant cell integrity and viability, leading to growth retardation, as well as reduced crop yield and quality. The UVR8 protein is identified as the specific UV-B receptor in plants. UVR8 signaling significantly contributes to UV-B adaptation responses and the establishment of UV-B tolerance. It is reported that in the absence of UV-B, UVR8 homodimers are primarily located in the cytoplasm. In the nucleus, the E3 Ubiquitin ligase COP1 (constitutively photomorphogenic 1) inhibits the activity of the transcription factor HY5 (elongated hypocotyl 5). Upon UV-B irradiation, UVR8 rapidly monomerizes and interacts with COP1 and WRKY36 in the nucleus. This complex prevents the degradation of HY5, and triggers UV-B regulated gene expression, resulting in plant adaptation and stress tolerance (Fernández et al., 2020). Researchers have also reported that UVR8 signaling induces the production of H_2_O_2_ and NO under UV-B irradiation. The resulting NO promotes stomatal closure, thereby limiting water loss and preventing cellular damage (Tossi et al., 2014).

A third candidate gene, *Sspon.04G0000680-2B* on chromosome 4B, encodes an R2R3 Myb transcription factor. Members of the R2R3 MYB family play important roles in plant growth, development, and flavonoid biosynthesis (Wang et al., 2019). Flavonoids contribute not only to pigmentation but also to UV protection, and R2R3 MYB proteins have been shown to regulate plant responses to light and protect against UV-induced damage (Jin et al., 2000; Hartmann et al., 2005). The basic helix-loop-helix (BHLH) factors and R2R3-MYB factors are known to work synergistically to direct the tissue-specific biosynthesis of flavonoids, while the ACE (ACGT-containing element)-binding factors (likely the basic region/leucine zipper, BZIP) and R2R3-MYB factors jointly contribute to conferring light responsiveness in plants (Hartmann et al., 2005). It has also been reported that in mutants of the Arabidopsis R2R3-MYB gene *AtMYB4*, the levels of sinapoyl esters in leaves are elevated. This mutant line exhibits greater tolerance to UV-B radiation compared to the wild type. This finding confirms that *AtMYB4* plays a critical role in regulating sinapoyl ester synthesis and the formation of UV-B protective mechanisms. *AtMYB4* appears to function as a repressor, particularly targeting the key gene encoding cinnamate-4-hydroxylase. By regulating the expression of this gene, *AtMYB4* negatively modulates sinapoyl ester synthesis under conditions without UV-B light exposure. When exposed to UV-B radiation, the expression of *AtMYB4* is downregulated, leading to an increase in sinapoyl ester production (Jin et al., 2000).

## 5 Conclusions

This study represents the first report of significant associations between single nucleotide polymorphisms (SNPs) and photosynthetic gas exchange parameters in sugarcane. Using genome-wide association analysis, the genetic basis of four photosynthetic gas exchange traits was investigated. A total of seven SNPs associated with intercellular CO_2_ concentration (Ci) across five quantitative trait loci (QTLs), and four SNPs associated with net photosynthetic rate (A) across four QTLs, were identified, encompassing eight genes in total.

Among these, three genes—*Sspon.02G0019170-1A*, *Sspon.02G0019190-3C*, and *Sspon.04G0000680-2B*—were identified as potential candidate genes influencing leaf photosynthesis based on functional annotations. These genes encode a WRKY transcription factor, the UV-B receptor UVR8, and an R2R3 Myb transcription factor, respectively, and were all associated with Ci on chromosomes 2A, 2C, and 4B.

The SNP markers and candidate genes identified in this study provide valuable resources for future functional studies and may facilitate marker-assisted breeding aimed at improving photosynthetic efficiency in sugarcane.

## Data Availability

The original contributions presented in the study are included in the article/**Supplementary Material**. Further inquiries can be directed to the corresponding author.
